# In 18F-positron emission tomography/computed tomography-guided precision radiotherapy for centrally located non-small cell lung cancer, tumor related atelectasis is a prognostic factor of survival

**DOI:** 10.3389/fonc.2022.898233

**Published:** 2022-07-28

**Authors:** Nan Wang, Yun Qiao, Yingqiu Song, Zheng Wang, Xia Li, Chengsen Liu, Ye Wang, Yu Wu, Rong He, Chenyu Wang, Yangwu Ren, Guang Li, Tianlu Wang

**Affiliations:** ^1^ Department of Radiotherapy, Liaoning Cancer Hospital & Institute, Cancer Hospital of China Medical University, Shenyang, China; ^2^ Department of Surgery, Liaoning Cancer Hospital & Institute, Cancer Hospital of China Medical University, Shenyang, China; ^3^ Department of Cerebral Surgery, Liaoning Cancer Hospital & Institute, Shenyang, China; ^4^ Department of Information Management, Liaoning Cancer Hospital & Institute, Shenyang, China; ^5^ Department of Epidemiology, School of Public Health, China Medical University, Shenyang, China; ^6^ Department of Radiotherapy, The First Hospital of China Medical University, Shenyang, China

**Keywords:** Centrally located NSCLC, Tumor related atelectasis, Prognostic factors, PET/CT, PSM

## Abstract

**Purpose:**

Tumor related atelectasis(TRA) is an essential factor affecting survival that can cause chest pain, cough, hemoptysis, chest tightness, dyspnea, and even death. In the current study, we explored the possible impact of TRA on survival in cancer patients and the guiding significance of 18F-positron emission tomography/computed(PET/CT) in radiotherapy for patients with atelectasis tumors.

**Methods:**

In this retrospective study, we analyzed the treatment model and survival of patients with centrally located non-small cell lung cancer(NSCLC) treated with radiotherapy at two medical centers between May 2005 and August 2019. We identified 152 eligible patients and used propensity score matching (1:1) to process the data to reduce confounding factors, data bias, and mal-distribution.

**Results:**

We used propensity scores created well-matched groups of 57 patients overall with or without TRA. The one-year survival rate of all patients was 71.9%, and the two-year survival rate was 33.3%. Compared to the atelectasis group, the overall survival (OS) of patients in the non-atelectasis group was significantly prolonged (25 months vs. 17 months, p = 0.004), as well as in the atelectasis recovery group (28 months vs. 14 months, p = 0.008). In multivariate analysis, non-atelectasis was closely correlated with favorable OS (HR, 1.804 (−2.840); 95% CI, 1.145–2.840; p = 0.011).

**Conclusion:**

PET/CT can accurately stage NSCLC and better guide the treatment of NSCLC complicated with atelectasis. Tumor-associated atelectasis in patients with centrally located NSCLC can lead to is a poor prognostic marker.

## Introduction

In cases where lung cancer is complicated with atelectasis, it is challenging to distinguish tumors from normal atelectatic lung tissue (NALT) by computed tomography (CT) images, mainly due to morphological changes in anatomy and tissue density. The atelectatic lung tissue has considerable overlap with the imaging of tumor tissue due to exudation, consolidation, and inflammatory reactions. Furthermore, both NALT and tumors tissue show high-density shadows on CT, so they are indistinguisable. However, determining the area of NALT and distinguishing it from the tumor tissue is very important to accurately delineate the radiotherapy target area and the treatment plan formulation (1, 2). The principle of 18F-positron emission tomography (18F-FDG PET) functional imaging is that the glucose metabolism rate of malignant tumor cells is higher than that of normal tissue cells. A previous study showed that FDG uptake in the atelectasis area is generally higher than normal lung parenchyma and lower than that in tumor tissue; since their concentration is different, the tumor tissue can be differentiated from the surrounding lung tissue (3). We previously reported (4) that PET/CT screening for non-small cell lung cancer (NSCLC) with atelectasis has significant advantages over traditional CT screening in guiding clinical treatment. PET/CT can make the clinical staging of NSCLC more accurate to design an appropriate treatment regimen, and improve the accuracy of target area when accompanied by NALT. Compared to CT, PET/CT can detect regional metastases and metastatic lymph nodes more effectively, and distinguish tumors and NALT more accurately, which improves the accuracy of radiotherapy target area (5, 6). Furthermore, according to the PET/CT images, three-dimensional conformal radiotherapy (3D-CRT) target area can be planned for the treatment of NSCLC, and reduce the exposure dose of the esophagus and spinal cord, thus facilitating an increase in radiotherapy dose (7). PET/CT-guided treatment can prolong the overall survival of patients with NSCLC (8).

The current TNM staging system considers TRA an adverse prognostic factor (9, 10), although a previous study has shown that TRA may prolong survival (11). Chen et al. has reported tumor-associated atelectasis had no significant effect on the survival of patients with superficial bronchial lung cancer (12). Moreover, Ou et al. has said that in stage IB NSCLC, tumor size determines hilar atelectasis or obstructive pneumonitis (13). The study by Coen et al. showed that partial or complete TRA might not affect overall survival (14). So far, the effect of TRA on the overall survival rate of radical radiotherapy for NSCLC has not been fully clarified. Therefore, we retrospectively analyzed whether TRA was a predictor for OS after PET/CT-guided definitive radiotherapy. Furthermore, we summarized the published clinical data in this setting and discussed the perspectives of PET/CT in radiation therapy for patients with centrally located NSCLC.

## Methods

### Study population and sources

We retrospectively reviewed NSCLC patients who underwent PET/CT examination (4, 15) at the Cancer Hospital of China Medical University and the First Hospital of China Medical University from May 2005 to August 2019. We reviewed patients’ electronic medical records and collected information such as age, gender, histological subtype, smoking history, radiographic, treatment, TRA, survival, state these variables. The inclusion criteria were patients with centrally located NSCLC who underwent PET/CT and received definitive radiation therapy. The complete basic information included whether the patients had a history of TRA and a clear re-expansion of the lungs after treatment? Our study used the eighth edition of the TNM staging standard endorsed by the International Association for the Study of Lung Cancer(IASLC) (16). The standard treatment for unresectable locally advanced NSCLC (stage IIB-IIIC) was concurrent radiotherapy and chemotherapy or sequential radiotherapy and chemotherapy. For selected patients with stage IV (oligometastatic: number of metastases ≤5), the standard treatment was systemic chemotherapy combined with local consolidation radiotherapy. We grouped patients according to the presence or absence of TRA. The evaluation of adverse reactions after radiotherapy and chemotherapy is based on the General Adverse Event Terminology Standard (version 4.0).

As this study was a retrospective study, the risk to the subjects was very small, and we obtained the exemption of informed consent.

### Propensity score matching (1:1)(PSM)

In the observational study, there are many data deviations and confounding variables due to various reasons. The propensity score matching method can make a more reasonable comparison between the experimental and control groups. We used IBM SPSS 25.0 software is used for propensity score matching, and used 1:1 nearest neighbor matching with 0.02 caliper width. The Chi-square test was used to evaluate the covariate balance before and after PSM in patients with TRA.

### Statistical methods

Baseline and clinicopathological characteristics were described in numbers (percentages) and compared by chi-square test and fisher’s exact test. Overall survival was calculated from the date of diagnosis to the date of death or the survivor’s last follow-up. The OS curves were generated by Kaplan-Meier method and comparison was done by log-rank test. We used Cox proportional hazard mode for Univariate and multivariate analysis, and we reported the results using hazard ratios (HRs) with 95% confidence intervals (CIs). All tests were two sided, and the results were considered signifcant at p values<0.05. Subgroup analyses according to the baseline characteristics were performed by drawing forest plots for overall survival using IBM SPSS (version 25.0; IBM Corporation, Armonk, NY) software and Stata MP 14 software (Stata Corp LLC, College Station, TX).

## Results

### Patients characteristics

**Figure 1:**
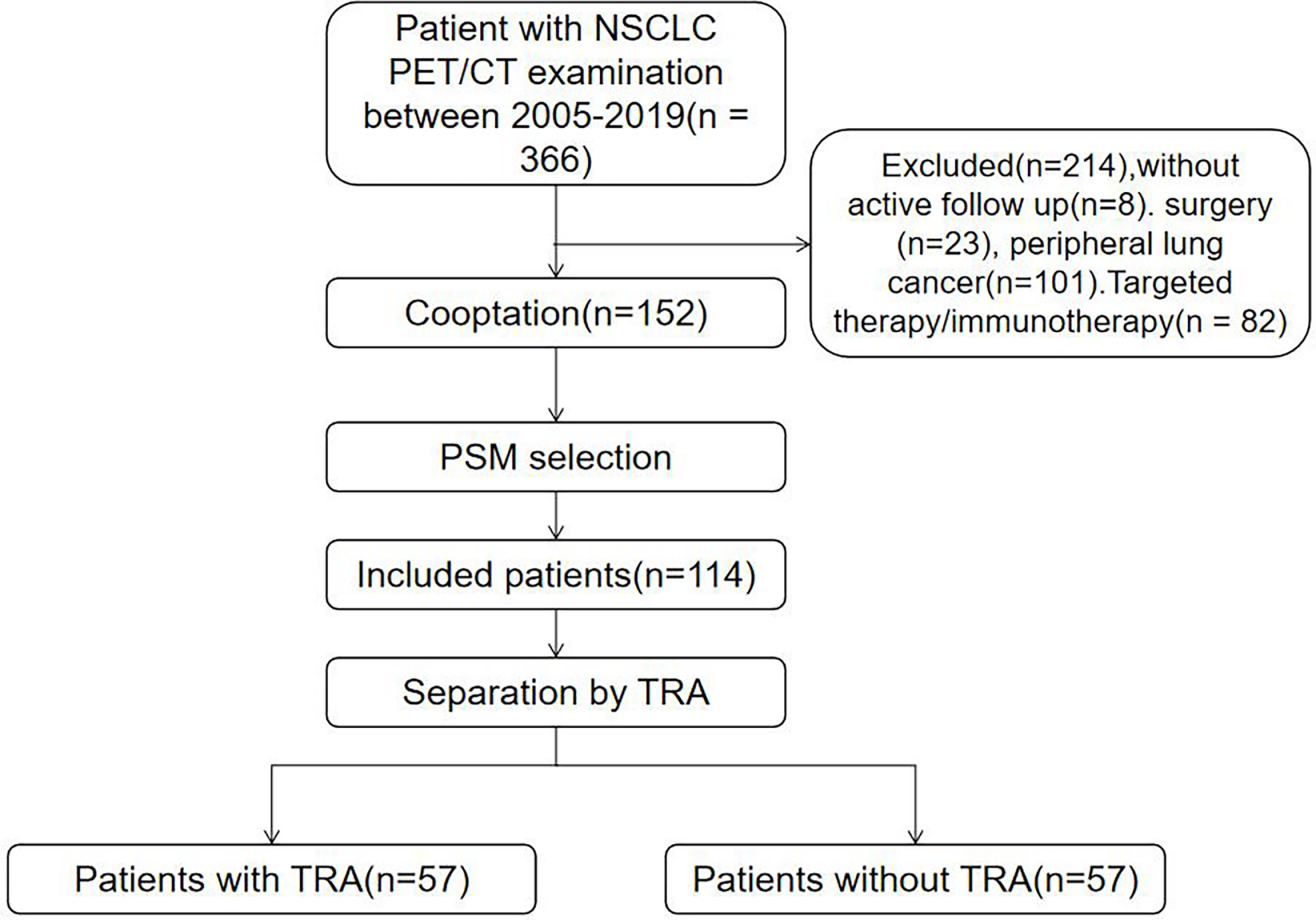
Flowchart depicting selection of the study population.

Of the 366 patients identified, 214 were excluded due to peripheral location of lung cancer, local surgery, incomplete data, or targeted therapy/immunotherapy; thus, a total of 152 eligible patients were included. The median follow-up period was 17 months. Of all patients, 34 patients are alive (22.4%), and 118 patients are deceased (77.6%). The causes of death included tumor-related complications (113 cases) and non-tumor complications (cerebrovascular events in 3 cases and myocardial infarction in 2 points). We created balanced groups of 57 patients with or without TRA through 1:1 PSM (**Figure 1**).

### Patient characteristics

**Table 1** shows that the baseline characteristics of the patients with and without TRA. Before PSM, the 152 matched patients (89 with TRA and 63 without TRA) included 120 men (78.9%) and 32 women (21.1%). A total of 15 cases (9.9%) were determined to have stage II, 109 cases (71.7%) stage III, and 28 cases (18.4%) stage IV. The median survival time was 17 months, the 1-year survival rate was 69.7%, and the 2-year survival rate was 30.9%. After PSM, the 114 matched patients (57 with TRA and 57 without TRA) included 88 men (77.2%) and 26 women (22.8%), with adenocarcinoma (25 cases, 21.9%), squamous cell carcinoma (77 cases, 67.5%), and other types (12 cases, 10.5%). In terms of patient stage, 11 cases (9.6%) were stage II, 80 cases (70.2%) were stage III, and 23 cases (20.2%) were stage IV. The median survival was 18.5 months, the 1-year survival rate was71.9%, and the 2-year survival rate was 33.3%.

**Table 1:** Baseline characteristics of patients.

characteristics	BEFORE PSM^a^			AFTER PSM^a^		
	No TRA (N=63)(%)	TRA (N=89)(%)	*P*	No TRA (N=57)(%)	TRA (N=57)(%)	*P*
**Age (years)**						
<65	36 (57.1)	56 (62.9)	0.473	35 (61.4)	35 (61.4)	1
≥65	27 (42.9)	33 (37.1)		22 (38.6)	22 (38.6)	
**Gender**						
Male	44 (69.8)	76 (85.4)	0.021	44 (77.1)	44 (77.1)	1
Famale	19 (30.2)	13 (14.7)		13 (22.9)	13 (22.9)	
**Smoking**						
No	33 (52.4)	62 (69.7)	0.03	29 (50.9)	38 (66.7)	0.087
Yes	30 (47.6)	27 (30.3)		28 (49.1)	19 (33.3)	
**Pathological pattern**						
squamous carcinoma	35 (55.6)	71 (79.8)	0.005	34 (59.6)	43 (75.4)	0.184
adenocarcinoma	18 (28.6)	13 (14.7)		15 (26.3)	10 (17.5)	
else	10 (15.9)	5 (5.6)		8 (14.1)	4 (7.1)	
**Weight loss**						
<5%	39 (61.9)	67 (75.3)	0.077	37 (64.9)	46 (80.7)	0.058
≥5%	24 (38.1)	22 (24.7)		20 (35.1)	11 (19.3)	
**KPS**						
>80	58 (92.1)	84 (94.4)	0.57	53 (93)	53 (93)	1^b^
≤80	5 (7.9)	5 (5.6)		4 (7)	4 (7)	
**T classification**						
T1-2	19 (30.2)	18 (20.2)	0.16	17 (29.8)	14 (24.6)	0.528
T3-4	44 (69.8)	71 (79.8)		40 (70.2)	43 (75.4)	
**N classification**						
N0-1	13 (20.6)	23 (25.8)	0.457	12 (21.1)	15 (26.3)	0.509
N2-3	50 (79.4)	66 (74.2)		45 (78.9)	42 (73.7)	
**Stages**						
IIb	6 (9.5)	9 (10.1)	0.159	5 (8.8)	6 (10.5)	0.233
IIIa	14 (22.2)	15 (23.8)		13 (22.8)	10 (17.5)	
IIIb	23 (36.5)	35 (55.6)		21 (36.8)	19 (33.3)	
IIIc	13 (20.6)	9 (14.3)		11 (19.3)	6 (10.5)	
IV	7 (11.1)	21 (23.6)		7 (12.3)	16 (28.1)	
**Chemotherapy**						
No	28 (44.4)	28 (31.5)	0.102	26 (45.6)	18 (31.6)	0.124
Yes	35 (55.6)	61 (68.5)		31 (54.4)	39 (38.4)	

a aThe PSM was performed using age, sex, which were subdivided according to the median values.

b .Fourfold table: sample size ≥ 40, 1 ≤ at least 2 theoretical frequencies < 5, using Fisher’s exact test;

n, number of cases/controls; PSM, propensity score matching.

### Analysis of prognostic factors

**Tables 2**, **3** show the results of univariate analysis before and after PSM, including the patient’s T stage of disease, whether TRA was present, and whether the lungs were re-expanded (all p < 0.1). **Figure 2** shows that in patients with centrally located NSCLC who did not undergo surgery and who underwent radical radiotherapy, patients with TRA had is a poor prognostic marker. For patients with TRA, re-expansion of the lungs represents a better prognosis, while non-TRA was associated with an apparent increase in the median OS (25 months vs. 17 months, p = 0.004). The OS of patients complicated with TRA was significantly improved after pulmonary dilatation (28 months vs. 14 months, p = 0.008) (**Figure 3**). Multivariate analysis showed that TRA was an independent factor predicting worse OS in patients with centrally located NSCLC receiving radical radiotherapy (p = 0.003) (**Tables 4**, **5**).

**Table 2:** Unificatory analyses of association between prognostic factors and overall survival (Before PSM).

characteristics	n	median survival time (months)	1-year os (%)	2-years os (%)	X^2^	*P*
**Age (years)**						
<65	92	19	69.6	26.1	2.55	0.576
≥65	60	22	70.0	38.3		
**Gender**						
Male	120	20	69.2	28.3	1.787	0.819
Famale	32	19	71.9	40.6		
**Smoking**						
No	95	19	64.2	28.4	0.741	0.363
Yes	57	21	78.9	35.1		
**Pathological pattern**						
squamous carcinoma	106	19	67.9	28.3	1.262	0.159
adenocarcinoma	31	27	71.0	38.7		
else	15	22	80.0	33.3		
**Weight loss**						
<5%	106	22	72.6	33.0	0.722	0.086
≥5%	46	14	63.0	26.1		
**KPS**						
>80	142	20	67.6	28.9	4.238	0.997
≤80	10	27	100.0	60.0		
**T classification**						
T1-2	37	23	81.1	35.1	0.407	0.021
T3-4	115	19	66.1	29.6		
**N classification**						
N0-1	36	24	83.3	38.9	1.402	0.184
N2-3	116	17	65.5	28.4		
**Stages**						
IIb	15	24	93.3	33.3	7.739	0.002
IIIa	29	27	82.8	44.8		
IIIb	58	21	69.0	32.8		
IIIc	22	12	54.5	9.1		
IV	28	12	57.1	28.6		
**Chemotherapy**						
No	56	15	62.5	32.1	0.062	0.42
Yes	96	22	74.0	30.2		
TRA						
No	63	24	76.2	38.1	2.593	0.003
Yes	89	17	65.2	25.8		
**Lung re-expansion**						
No	66	14	63.6	22.7	3.753	0.006
Yes	23	28	69.6	34.8		

**Table 3:** Unificatory analyses of association between prognostic factors and overall survival (After PSM).

characteristics	n		median survival time (months)	1-year os (%)	2-years os (%)	X^2^	*P*
**Age (years)**							
<65	70		20	72.9	30.0	0.907	0.584
≥65	44		22	70.5	38.6		
**Gender**							
Male	88		21	72.7	30.7	1.221	0.912
Famale	26		19	69.2	42.3
**Smoking**							
No	67		19	65.7	29.9	0.887	0.261
Yes	47		27	80.9	38.3
**Pathological pattern**							
squamous carcinoma	77		19	70.1	29.9	1.696	0.098
adenocarcinoma	25		31	76.0	44.0
else	12		22	75.0	33.3
**Weight loss**							
<5%	83		22	75.9	34.9	0.354	0.375
≥5%	31		16	61.3	29.0
**KPS**							
>80	106	20		69.8	31.1	3.294	0.965
≤80	8	27		100.0	62.5
**T classification**							
T1-2	31	25		83.9	35.5	0.089	0.009
T3-4	83	19		67.5	32.5
**N classification**							
N0-1	27	22		85.2	37.0	0.218	0.363
N2-3	87	19		67.8	32.2
**Stages**							
IIb	11	22		90.9	27.3	5.986	0.015
IIIa	23	27		87.0	47.8
IIIb	40	24		70.0	35.0
IIIc	17	13		58.8	11.8
IV	23	14		60.9	34.8
**Chemotherapy**							
No	44	16		61.4	31.8	0.074	0.202
Yes	70	24		78.6	34.3
**Atelectasis**							
No	57	25		75.4	38.6	1.421	0.004
Yes	57	17		68.4	28.1
**Lung re-expansion**							
No	41		14	65.9	22.0	3.882	0.008
Yes	16		28	75	43.8

**Figure 2:**
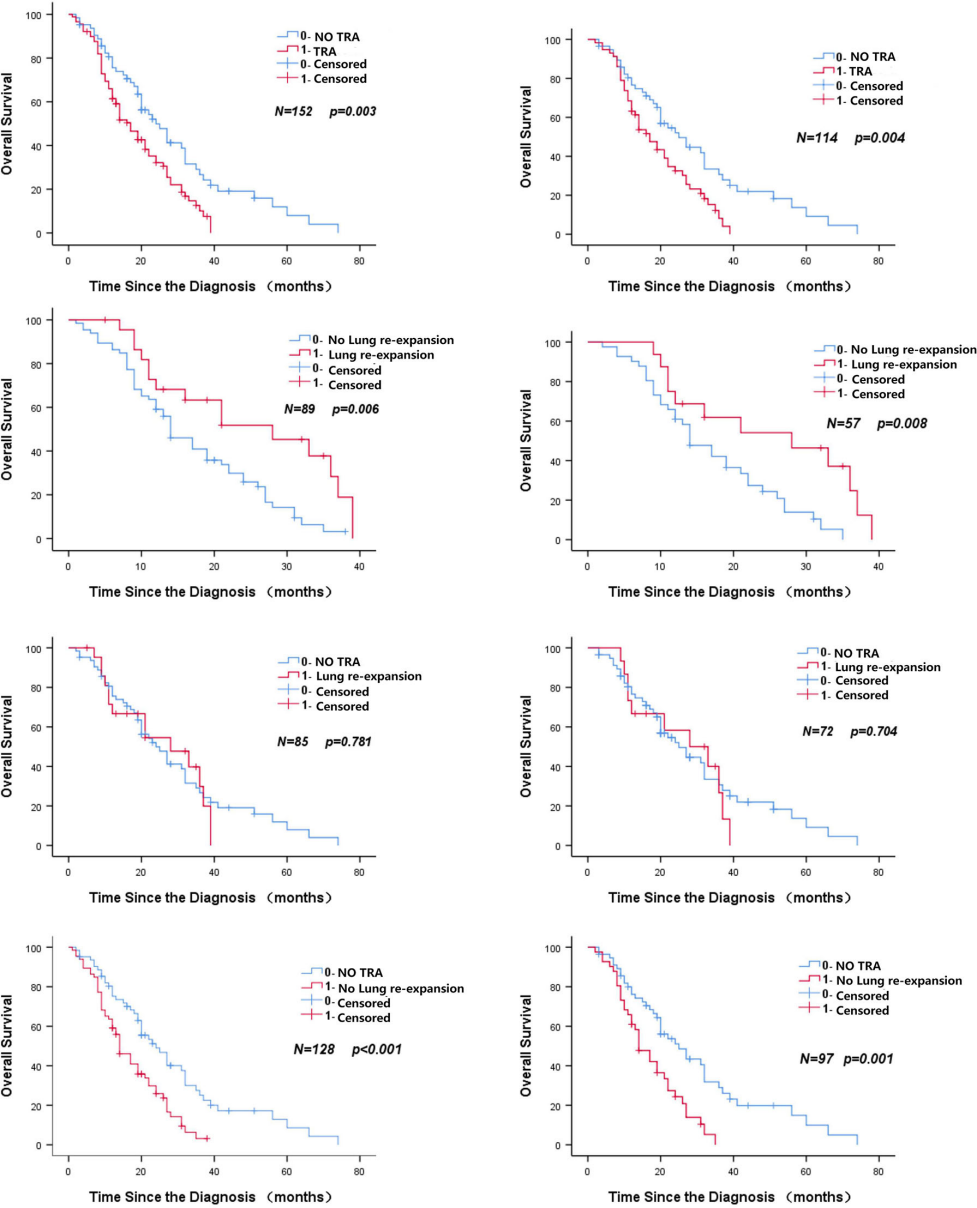
Kaplan–Meier curve of overall survival for atelectasis or non-atelectasis patients. (LEFT BEFORE PSM RIGHT AFTER PSM).

**Figure 3:**
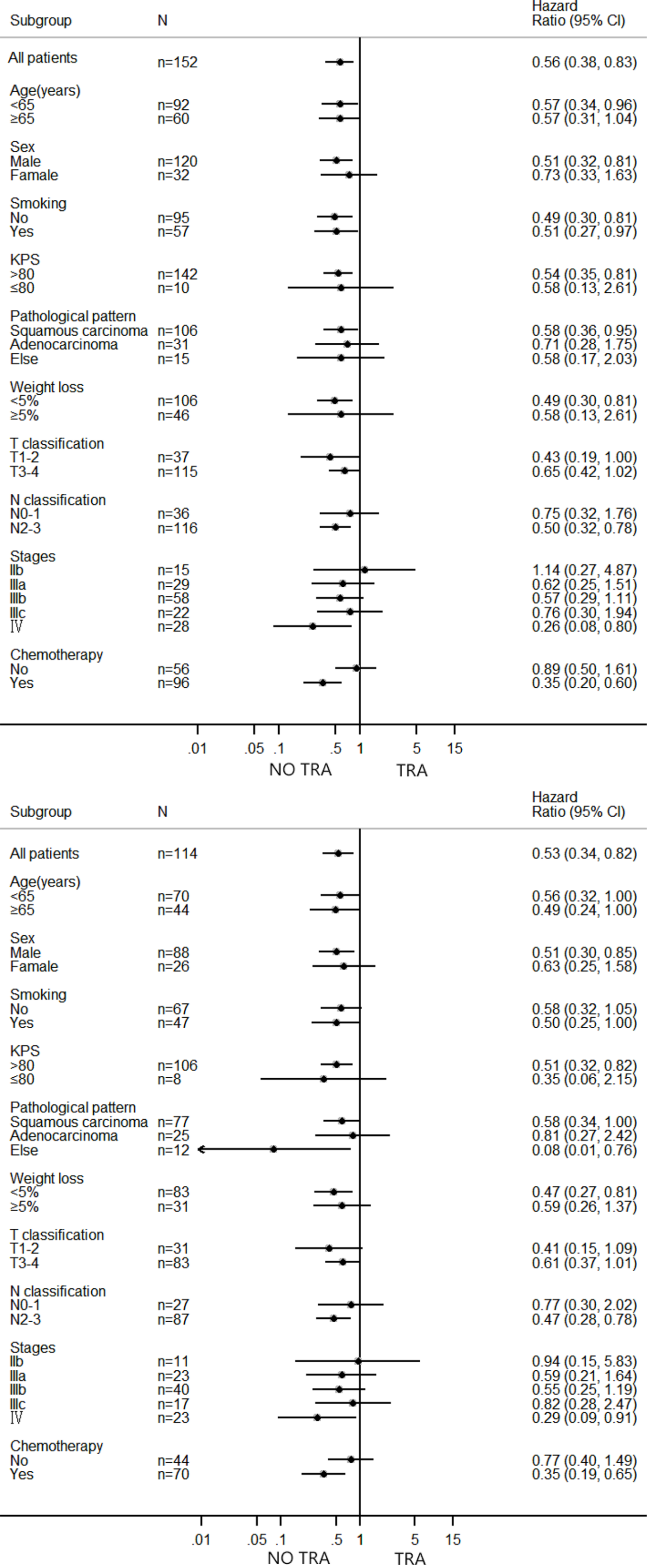
Patients were included in the OS subgroup analysis. (LEFT BEFORE PSM RIGHT AFTER PSM).

**Table 4:** Multivariate analysis between prognostic factors and overall survival (Before PSM).

Facter	B	Sx	*P*	HR (95%CI)
T1~2 vs. T3~4	.200	.244	.412	1.221 (0.758-1.969)
No Atelectasis vs. Atelectasis	.580	.205	.005	1.786 (1.196-2.667)
Stages(IIb/IIIa/IIIb/IIIc/IV)	.240	.078	.002	1.272 (1.091-1.482)

**Table 5:** Multivariate analysis between prognostic factors and overall survival (After PSM).

Facter	B	Sx	*P*	HR (95%CI)
T1~2 vs. T3~4	.408	.282	.148	1.504 (.865-2.614)
No Atelectasis vs. Atelectasis	.590	.232	.011	1.804 (1.145-2.840)
Stages(IIb/IIIa/IIIb/IIIc/IV)	.200	.089	.025	1.222 (1.026-1.455

## Discussion

As far as we know, this is the most extensive retrospective study to date, aiming to evaluate the predictive value of TRA in patients with centrally located NSCLC treated with radiotherapy guided by PET/CT. We determined that TRA had a significantly negative prognostic indicator of OS in centrally located NSCLC after PET/CT-guided definitive radiotherapy. Moreover, lung re-expansion was a favorable independent prognostic factor in the TRA subgroup. Nonetheless, we found interesting results. There was no statistical difference in overall survival between the lung re-expansion and the no TRA group. The study further proves that TRA is a significant prognostic factor in patients with centrally located NSCLC, and we infer that the disease symptoms are improved after lung re-expansion. TRA is an important factor affecting survival that can cause chest pain, cough, hemoptysis, chest tightness, dyspnea, and even death. In addition, lung re-expansion improves tumor hypoxia and is increases chemoradiotherapy sensitivity; as we know, tumor hypoxia is a significant cause of tumor radiation resistance (6). Another factor related to prolonging survival is the control of infection and the improved immunity in lung re-expansion (6). Our study suggests that patients with atelectasis can be considered for radical radiotherapy under FDG-PET/CT guidance. We assume that mid/post-treatment lung re-expansion analysis is worth exploratory and would require further confirmation in future studies. After multivariate analysis, both clinical stage and TRA were independent prognostic factors.

Two main questions arose from the studies reported: First, is the T-classification in TNM staging applicable to patients with TRA? Based on anatomical image CT, the treatment plan and target area delineation developed for atelectasis patients should be done with functional imaging PET methods that includes multiple confounders. For example, the determination of tumor boundaries, disseminated lesions in unambiguous lung tissue, mediastinal lymph node overlap with TRA, and distant occult metastases. TRA, the most frequent thoracic complication in central lung cancer, has a considerable impact on the quality of life, making lung cancer patients’ treatment more difficult. 18F-FDG PET/CT has good sensitivity to defect metastasis in systemic soft tissue, viscera (lungs, liver, adrenal gland, etc.), distant lymph nodes, and bones, and Several meta-analyses emphasized its role in accurate staging[17, 18]. Furthermore, an accurate depiction of the target volumes can prevent the omission of the radiotherapy target area. The wrong delineation of the gross tumor volume (GTV) (detectable tumor) is a common mistake. The incorrect tracing of target areas will lead to insufficient radiotherapy dose and reduce local control rate. With the development of 3D radiotherapy technology, we can delineate accurate treatment plan based on three-dimensional reconstruction of CT images, evaluate the treatment volume, and overall scheme, further optimize the radiation dose in the tumor area, reach the radical cure standard, and reduce the radiation effect on normal tissues (19). Secondly, When lung cancer is complicated with TRA, it is difficult to determine the tumor area only by anatomical images such as CT, which will inevitably affect the treatment results. In contrast to previous studies (11–14), this study retrospective study included cases treated using functional imaging PET/CT guidance to develop treatment plan, outline target areas, and establish radiotherapy plans. Our previous study (8) showed that radiation treatment guided by PET-CT had a better outcome in the similar stage than radiation treatment without PET-CT guidance.

The standard treatment for unresectable locally advanced NSCLC (stage IIB-IIIC) in our study was concurrent radiotherapy and chemotherapy or sequential radiotherapy and chemotherapy.

In our study, the standard of care for most patients was concurrent radiotherapy and chemotherapy or sequential radiotherapy and chemotherapy. The definitive radiation treatment for unresectable locally advanced NSCLC (stage IIB-IIIC) is an equivalent dose of 60–66 Gy/30f(BED). For patients (in selected cases) with stage IV (oligometastatic), the standard treatment in our study was systematic chemotherapy combined with local consolidation radiotherapy (50-60 Gy). The purpose of consolidation radiation is to improve the control rate of the primary tumor area and prevent distant metastasis. In our previous study, local consolidation treatment for patients with stage IV oligometastatic NSCLC can improve patient outcomes to some extent (20). Local consolidation treatment may cause modest toxicity, but can improve overall survival. Using PET/CT pretreatment characterization of tumors using PET/CT may help patients find the most suitable treatment, accurately customize radiotherapy plans, and select chemotherapeutics. Incorrect GTV depiction may lead to insufficient dose to the tumor, leading to local control failure and/or increased toxicity due to radiation of normal tissue. However, it may be challenging to depict GTV entirely based on planned CT scans and is affected by differences within and between observers (4). When tumors and surrounding tissues with similar densities appear on CT images, their contrast is limited, making it more challenging to determine GTV. Especially in the presence of TRA (lung collapse or closure), mediastinal invasion, and GTV should include mediastinal lymph nodes. The inclusion of FDG-PET images in radiotherapy planning has been shown to reduce intra observer and inter-observer variability and improve the clarity of GTV (21). In addition, there is a good correlation between the tumor size determined by resected pathological specimens and the tumor size in preoperative PET/CT images (22). In the use of FDG-PET/CT images to guide radiation treatment planning, using the 3D-CRT/IMRT radiotherapy technique, we select some stage IV NSCLC high palliative chest consolidation measures. Dose escalation is carried out through integrated enhancement technology and will not exceed the dose limit of normal organs and tissues. Similar strategies were used with both TRA and no TRA, whereby the prescribed dose was increased to residual tumor volumes. In contrast to previous studies, the same approach was used in our patients: Patients underwent FDG-PET/CT-guided 3D-CRT/IMRT radiotherapy, with dose escalation to residual tumor areas after 14–20 fractions of radiotherapy, combined with (concurrent or sequential) chemotherapy or solely radiotherapy. There was no significant difference between the TRA and no TRA groups in terms of toxic side effects after chemoradiotherapy, both of which were below grade 3. Furthermore, our previous results (5) showed that this PET CT guided treatment approach was superior to the outcomes of patients based on CT-guided radiotherapy regimens during the same period at the same institution. This could be related to collapse and hypoxia of lung parenchyma. Atelectasis and obstructive pneumonia caused by bronchial compression or involvement are important causes of death in patients with lung cancer (6). Hypoxia induces increased cellular glucose uptake, and proximal tumors in the airway often lead to atelectasis and bronchial obstruction.

This study is a retrospective study. The sample size is relatively small and there is heterogeneity between cases, which is prone to selection bias and recall bias. In addition, due to the large time span, this study lacks corresponding clinical data on the impact of driver gene positive related targeted therapy and immunotherapy on the overall survival of oligometastatic NSCLC patients in recent years, and does not study the cause of death of patients.

Taken together, our results demonstrate that PET/CT guided radiation treatment plan has distinct advantages in guiding patients with atelectasis to 3D-CRT/IMRT radiotherapy. Moreover, it can accurately stage lung cancer, develop a standardized, accurate, and effective treatment plan based on precise staging, and better complete radiotherapy by mapping the target area. TRA and T stage are strongly correlated with unfavorable prognosis in patients with centrally located NSCLC definitive radiotherapy under the guidance of PET/CT. Active treatment of TRA can prolong the survival of patients, while lung re-expansion can improve the prognosis of atelectasis patients. Since the small number of patients with lung re-expansion after chemoradiotherapy limits its applicability, we caution that post/mid-treatment lung re-expansion analysis should be considered exploratory until further validation in larger cohorts.

## Data availability statement

The original contributions presented in the study are included in the article/supplementary material. Further inquiries can be directed to the corresponding author.

## Ethics statement

The ethics committees approved the retrospective protocol of the current study of Cancer Hospital of Chinese Medical University and The First Hospital of China Medical University, which was conducted in accordance with the Declaration of Helsinki. Since all data are anonymous, the requirement of informed consent is exempted.

## Author contributions

Conceptualization: TW and GL; data curation: NW, YS, and YQ; formal analysis: NW, YS, and ZW; statistical analysis: YS and ZW; validation: NW; visualization: YR and C-YW; writing the original draft: NW, YS, and YQ; draft review & editing: all authors.; modify and polish: all authors; funding acquisition: TW. All authors read and approved the final manuscript.

## Funding

The Liaoning Province Natural Science Foundation [2020-ZLLH-47][2020-MS-065], Medical Industry Cross Joint Fund of Liaoning Province (Dalian University of Technology)[2021-YGJC-02], Liaoning Province Key Area Joint Open Fund [2019-KF-01-01], Tumor Mass spectrometry Center project[ZP202013][ZP202008], Key Laboratory of Tumor Radiosensitization and Normal Tissue Radioprotection Project of Liaoning Province (2018225102), Shenyang Major Scientific Research Projects (19–112–4–090).

## Conflict of interest

The authors declare that the research was conducted in the absence of any commercial or financial relationships that could be construed as a potential conflict of interest.

## Publisher’s note

All claims expressed in this article are solely those of the authors and do not necessarily represent those of their affiliated organizations, or those of the publisher, the editors and the reviewers. Any product that may be evaluated in this article, or claim that may be made by its manufacturer, is not guaranteed or endorsed by the publisher.
